# Anti-Inflammatory Effects of Curcumin via the Nrf2-cGAS-STING-NF-κB Pathway in MH7A Rheumatoid Arthritis Fibroblast-like Synoviocytes

**DOI:** 10.3390/biomedicines14030611

**Published:** 2026-03-09

**Authors:** Luyao Li, Tong Shen, Zhen Li, Qianyu Guo, Quanhai Pang

**Affiliations:** 1 College of Veterinary Medicine, Shanxi Agricultural University, Jinzhong 030801, China; 16634253443@163.com (L.L.); shentong0613@163.com (T.S.); 2 College of Basic Medical Sciences, Shanxi University of Chinese Medicine, Jinzhong 030619, China; lz88@sxtcm.edu.cn; 3 Department of Rheumatology, Shanxi Bethune Hospital, Shanxi Academy of Medical Sciences, Taiyuan 030032, China

**Keywords:** rheumatoid arthritis, curcumin, NRF2-cGAS-STING-NF-κB, synovial fibroblasts

## Abstract

**Highlights:**

**What is the main finding?**

- Curcumin (CUR) can effectively inhibit the inflammatory response of synovial fibroblasts by activating the expression of NRF2 and subsequently suppressing the cGAS-STING-NF-κB signaling pathway.

**What is the implication of the main finding?**

- This study provides a new molecular mechanism target for curcumin in the treatment of RA and offers a theoretical basis for the intervention of autoimmune diseases with natural products.

**Abstract:**

**Background:** Abnormal activation of the NRF2-cGAS-STING-NF-κB pathway can trigger an inflammatory cascade in rheumatoid arthritis (RA). Curcumin (CUR), a polyphenolic compound extracted from turmeric, possesses anti-inflammatory activity, but whether it can modulate this pathway to ameliorate RA remains unclear. This study aims to elucidate whether CUR inhibits the inflammatory response in synovial fibroblasts (MH7A) by suppressing the NRF2-cGAS-STING-NF-κB signaling cascade. **Methods:** An RA inflammatory model was constructed by stimulating MH7A cells with 20 ng/mL tumor necrosis factor (TNF). Groups included a control group, a model group, a methotrexate positive control group [MTX(methotrexate), 10 μmol/L], and curcumin treatment groups at varying concentrations (10–100 μmol/L). Cell viability was assessed using the CCK-8(Cell Counting Kit-8) assay. Cell migration and invasion capabilities were evaluated via scratch wound healing and Transwell assays, respectively. Apoptosis was detected by flow cytometry. mRNA and protein expression levels of NRF2(Nuclear factor erythroid 2-related factor 2), cGAS(cyclic GMP-AMP synthase), STING(stimulator of interferon genes), and NF-κB(nuclear factor kappa-light-chain-enhancer of activated B cells) were measured using qRT-PCR and Western blot, respectively. Protein localization was determined by immunofluorescence. **Results:** Compared to the model group (TNF-induced), the cell migration rate in the curcumin (CUR) groups was significantly decreased (*p* < 0.001), with a particularly marked reduction observed at a concentration of 50 μmol/L. Furthermore, as the concentration of curcumin increased, cell invasion capacity showed a significant dose-dependent decline. The apoptosis rate also significantly decreased with increasing curcumin concentrations, demonstrating a clear concentration-dependent effect. Mechanistically, curcumin treatment significantly upregulated the expression of NRF2 and inhibited the activation of its downstream cGAS-STING-NF-κB signaling pathway. Specifically, both mRNA and protein expression levels of NRF2 were markedly elevated (*p* < 0.001), while the mRNA and protein levels of cGAS, STING, and NF-κB were all significantly reduced (*p* < 0.001). **Conclusions:** Curcumin (CUR) can effectively inhibit the inflammatory response of synovial fibroblasts by activating the expression of NRF2 and subsequently suppressing the cGAS-STING-NF-κB signaling pathway. This study provides a new molecular mechanism target for curcumin in the treatment of RA and offers a theoretical basis for the intervention of autoimmune diseases with natural products.

## 1. Introduction

Rheumatoid arthritis (RA) is an immune-mediated, inflammatory, chronic, progressive, systemic autoimmune disease [1]. In the middle-aged female population, the incidence of RA is relatively high. Globally, the incidence of RA is approximately 1%. In addition to affecting normal quality of life, RA also has a disability rate of about 30% and can even impact normal life expectancy [2]. Its core pathological feature is persistent inflammation of the synovial tissue, leading to progressive destruction of articular cartilage and bone [3,4]. The activation of synovial fibroblasts (FLS) is particularly crucial in this process. Activated FLS exhibit enhanced proliferation, migration, and invasion capabilities, along with resistance to apoptosis [5], displaying “tumor-like” characteristics that directly contribute to cartilage erosion and bone destruction. Current clinical treatments for RA, such as non-steroidal anti-inflammatory drugs (NSAIDs), disease-modifying antirheumatic drugs (DMARDs) [6], and biologics, are often associated with side effects like gastrointestinal damage and immunosuppression with long-term use, and have limited efficacy in reversing the invasive phenotype of FLS [7,8]. Therefore, exploring new therapeutic strategies that can safely and effectively intervene in the malignant behavior of RA-FLS through multi-target mechanisms is of significant importance.

In this context, the polyphenolic compound extracted from turmeric—curcumin—has garnered significant attention due to its potent anti-inflammatory and immunomodulatory activities. Clinical studies have demonstrated that curcumin exhibits favorable therapeutic effects in RA [9,10,11]. Nevertheless, the precise molecular network through which it acts, particularly whether it can directly modulate key pathogenic signaling axes in RA-FLS, remains incompletely elucidated. This knowledge gap, to some extent, constrains the precision of its clinical application and hinders the advancement of rational and safe medication practices.

NRF2 is a key transcription factor that regulates oxidative stress and inflammatory responses [12,13] Upon activation, it can inhibit the NF-κB signaling pathway [14], thereby exerting anti-inflammatory effects. Recent studies have found that sustained oxidative stress in the pathological environment of rheumatoid arthritis (RA) can lead to intracellular DNA damage and mitochondrial DNA (mtDNA) leakage, which abnormally activates the cytosolic DNA sensor cGAS and its adaptor protein STING. The activated cGAS-STING pathway not only promotes the production of type I interferons but also extensively cross-talks with the classic pro-inflammatory transcription factor NF-κB signaling. Together, they drive the inflammatory response, migration, and invasion of fibroblast-like synoviocytes (FLS) [15], constituting a key driving force for joint destruction in RA. Further research has revealed that NRF2 deficiency can exacerbate DNA damage and enhance the activation of the cGAS-STING pathway, suggesting that NRF2 may function upstream of the cGAS-STING-NF-κB signaling axis, playing a negative regulatory role. Curcumin, as a known NRF2 activator and NF-κB inhibitor, has been extensively demonstrated to inhibit the proliferation of RA-FLS, achieving therapeutic effects in RA [16]. However, whether curcumin can curb the activation of RA-FLS by modulating the complete “NRF2-cGAS-STING-NF-κB” signaling cascade network remains unknown.

Based on this, we propose the scientific hypothesis: curcumin may inhibit the excessive activation of the cGAS-STING-NF-κB signaling axis by activating NRF2, thereby suppressing the proliferation, migration, and invasion of RA-FLS while promoting their apoptosis. To test this hypothesis, this study employed the human RA synovial fibroblast cell line MH7A as an in vitro model, using tumor necrosis factor (TNF) [17] to simulate inflammatory stimulation. We systematically evaluated the effects of curcumin on the malignant phenotype of the cells and focused on investigating the core mediating role of the “NRF2-cGAS-STING-NF-κB” cascade pathway. This study aims to provide new experimental evidence and theoretical foundations for elucidating the deep molecular mechanisms of curcumin in treating RA.

## 2. Materials and Methods

### 2.1. Experimental Materials

#### 2.1.1. Experimental Cells

The human rheumatoid arthritis fibroblast-like synoviocyte cell line (MH7A cell strain) was purchased from Saiaosi Biotechnology (Wuhan China), with batch number: CL-474h. Cells were cultured in DMEM high-glucose medium supplemented with 10% FBS and 1% penicillin-streptomycin mixture, and maintained in a constant temperature incubator at 37 °C with 5% CO_2_ and saturated humidity.

#### 2.1.2. Drugs and Main Reagents

Curcumin (Shanghai, China, Macklin, CAS No.: 458-37-7), Metho-trexate tablets (Shanghai, China, Shangyao Xinyi Pharmaceutical Co., Ltd., Batch No.: 197221004), Dimethyl sulfoxide (DMSO) (Macklin, CAS No.: 1187431-43-1); Total RNA Extraction Kit, SDS-PAGE Gel Preparation Kit, BCA Protein Concentration Assay Kit, DAB Chromogenic Kit (Beijing, China, Solarbio Science & Technology Co., Ltd., Cat. Nos.: G1371, R1200, P1200, PC0020, DA1010); RT mix with DNase (AII-in-One), Universal SYBR Green qPCR Supermix (US EVERBRIGHT, San Francisco, CA, USA, Cat. Nos.: R2020L, S2024L); Tri-color Pre-stained Protein Marker (Jinan, China, Scikjie Biotechnology Co., Ltd., Cat. No.: EC1019-A); Rabbit Anti-GAPDH Antibody (Beijing, China, Beijing Biosynthesis Biotechnology Co., Ltd., Cat. No.: bsm-33033M); NRF2 Antibody, cGAS Antibody, STING Antibody, NF-κB Antibody (Wuhan, China, Wuhan Sanying Antibody Co., Ltd., Cat. Nos.: 16396-1-AP, 26416-1-AP, 19851-1-AP, 80979-1-AP); Goat Anti-Rabbit IgG Antibody, Goat Anti-Rat IgG Antibody (Beijing, China, Solarbio Science & Technology Co., Ltd., Cat. Nos.: SE134, SE132); DMEM Medium (Gibco, USA, Grand Island, NY, USA), Fetal Bovine Serum (FBS) (Lanzhou, China, cellmax, Cat. No.: SA211), Penicillin-Streptomycin Dual Antibiotic (Beijing, China, Solarbio, Cat. No.: P1400), Phosphate(Serhal, Lwin et al. 2020 [1]) Buffered Saline (PBS) (Solarbio, Cat. No.: P1020), Trypsin (Solarbio, Cat. No.: T1300), Tumor Necrosis Fac-tor-α (TNF) (Suzhou, China, Suzhou Jin’an Protein Technology Co., Ltd., Cat. No.: CG90), CCK-8 Solution (Shanghai, China, Beyotime Biotechnology, Cat. No.: C0038), RIPA Lysis Buffer (Beyotime Biotechnology, Cat. No.: P0013B), Annexin V-FITC/PI Apopto-sis Detec-tion Kit (Liaoning, China, Melunbio, Cat. No.: MA0220), AF488-labeled Goat Anti-Rabbit IgG (H + L), AF488-labeled Goat Anti-Mouse IgG (H + L) (Beyotime Biotechnology, Cat. Nos.: A0423, A0428), Antifade Mounting Medium (with DAPI), Goat Serum (Sterile Fil-tered), Triton X-100 (Solarbio, Cat. Nos.: S2110, S9070, T8200). CCK-8 Cell Proliferation And Cytotoxicity Assay Kit (Solarbio, Cat. Nos.: CA1210).

#### 2.1.3. Instruments

Biological microscope (Chongqing Aote Optical Instrument Co., Ltd., Chongqing, China, Model DM2500 LED); Navios flow cytometer (Beckman Coulter, Inc., Brea, CA, USA); Real-time fluorescent quantitative PCR instrument (Bio-Rad Laboratories, Inc., Hercules, CA, USA, Model Light Cycler 96); UV-Vis spectrophotometer (Shanghai Mapada Instruments Co., Ltd., Shanghai, China, Model UV-1800PC); ChemiDoc™ imaging system (Bio-Rad Laboratories, Inc., Hercules, CA, USA, Model 734BR4160).

### 2.2. Experimental Methods

#### 2.2.1. Cell Culture and Model Establishment

MH7A cells were seeded in DMEM medium supplemented with 10% fetal bovine serum and 1% penicillin-streptomycin, and cultured in a humidified incubator at 37 °C with 5% CO_2_. When cells reached 80–90% confluence, they were detached using 0.25% trypsin-EDTA and prepared as a single-cell suspension for subculture. Cells from passages 6–10 were used for all experiments.

#### 2.2.2. Cell Grouping and Treatment

The model group consisted of MH7A cells treated with 20 ng/mL TNF. The drug-treated groups were MH7A cells co-treated with 20 ng/mL TNF and curcumin at concentrations of 10, 25, 50, 75, or 100 μmol/L. The positive drug control group consisted of MH7A cells co-treated with 20 ng/mL TNF and 10 μmol/L methotrexate (MTX).

#### 2.2.3. Cell Viability Assay (CCK-8)

MH7A cells in the logarithmic growth phase were seeded in a 96-well plate at a density of 1.0 × 10^5^ cells/mL. Five intervention groups with different concentrations of curcumin were set up: 10, 25, 50, 75, and 100 μmol/L. After allowing the cells to adhere and grow for 24 h, culture medium containing the corresponding drug was added according to the experimental groups, followed by incubation at 37 °C with 5% CO_2_. Cell viability was assessed using the CCK-8 assay at 12, 24, and 48 h post-treatment, and the cell proliferation inhibition rate was calculated.

Experimental principle: The reagent contains a water-soluble tetrazolium salt, WST-8. In living cells, dehydrogenases within the mitochondria, facilitated by an electron carrier (1-Methoxy PMS), reduce WST-8 to an orange-yellow formazan product. The amount of formazan produced is directly proportional to both the number of viable cells and their metabolic activity.

Consequently, the greater the cytotoxicity, the higher the number of dead cells, or the more severely proliferation is inhibited, the lighter the color. Conversely, the more viable cells present and the faster they proliferate, the deeper the color becomes. Detection is performed by measuring the absorbance (OD value) at a wavelength of 450 nm using a microplate reader, which indirectly reflects the quantity of living cells.

#### 2.2.4. Cell Migration and Invasion Assays

Cell Migration Assay (Wound Healing Assay)

Cells were seeded in 6-well plates at a density of 1 × 10^7^ cells/well and cultured until a confluent monolayer formed (approximately 90% confluency). A sterile 1000 μL pipette tip was used to create a uniform scratch through the cell monolayer along a pre-marked line. The wells were gently washed with PBS several times to remove detached cells and debris. After returning the plates to the CO_2_ incubator for 1 h, fresh serum-free medium was added. Subsequently, 2 mL of curcumin solutions at different concentrations were added to the corresponding wells, and the plates were placed back into the CO_2_ incubator for continued culture. The scratch wounds were observed periodically over several hours. After 24 h of incubation, images were captured. Finally, the results were analyzed.

##### Transwell Invasion Assay

This experiment was performed to assess the effect of different concentrations of curcumin on the invasive ability of MH7A cells. Cells were cultured to the logarithmic growth phase, trypsinized, and resuspended in serum-free medium at a density of 5 × 10^5^ cells/mL. In the lower chamber of the Transwell plate, 500 μL of complete medium containing 30% FBS was added. The Transwell insert was then placed, and 200 μL of the serum-free cell suspension was carefully added to the upper chamber without touching the membrane. Care was taken to avoid bubble formation between the insert and the lower chamber medium. The entire setup was incubated in a CO_2_ incubator at 37 °C for 24 h. After incubation, non-invaded cells and medium from the upper chamber were removed, and the insert was gently washed with PBS. Cells that had invaded through the membrane were fixed with 4% paraformaldehyde for approximately 15 min, washed again with PBS, and then stained with crystal violet for visualization. After staining, excess dye was washed off with distilled water. Images were captured, and data were processed for analysis.

#### 2.2.5. Cell Apoptosis Detection (Flow Cytometry)

The culture medium from the cell culture flask was aspirated into a nuclease-free centrifuge tube. Cells were washed once with PBS, and the PBS was collected back into the same tube. An appropriate amount of trypsin (without EDTA) was added, followed by incubation at room temperature for 20 min. Complete culture medium was added to terminate digestion. The digested cells were transferred to the centrifuge tube containing the collected medium and centrifuged for 5 min. The supernatant was discarded, and the cell pellet was collected. Cells were resuspended in 1 mL of culture medium and counted. Approximately 1 × 10^6^ resuspended cells were aliquoted and centrifuged for 5 min. The supernatant was discarded, and the pellet was resuspended in PBS and centrifuged again. After discarding the supernatant, cells were gently resuspended in 195 μL of Annexin V-FITC binding buffer. Then, 5 μL of Annexin V-FITC was added, mixed well, followed by the addition of 10 μL of propidium iodide (PI) staining solution. The mixture was gently vortexed and incubated at room temperature in the dark for 10–20 min. Samples were then analyzed using a flow cytometer.

#### 2.2.6. Real-Time Quantitative PCR (RT-qPCR) Detection of mRNA Expression of NRF2, cGAS, STING, and NF-κB in Each Group

After 24 h of cell culture in each experimental group, total RNA was extracted. The purity and concentration of total RNA were measured using a micro-volume spectrophotometer, and the RNA samples were stored at −80 °C for subsequent use. According to the instructions of the HiScript III RT SuperMix for qPCR kit, RNA was reverse transcribed into cDNA. The mRNA expression levels of NRF2, cGAS, STING, and NF-κB in each group were detected using quantitative real-time PCR (qRT-PCR). A 20 µL reaction system was prepared as follows: 10 µL of 2× Realtime PCR Super mix, 2.0 µL of cDNA template, 0.5 µL each of forward and reverse primers, and 7.0 µL of ddH_2_O. The PCR amplification program consisted of: initial denaturation at 94 °C for 5 min; 40 cycles of 94 °C for 30 s, 60 °C for 30 s, and 72 °C for 30 s; and final extension at 72 °C for 5 min. Each target was assayed in triplicate. The relative mRNA expression levels of NRF2, cGAS, STING, and NF-κB in each group were calculated using the 2^−ΔΔCT^ method. Real-Time Quantitative PCR (RT-qPCR) Detection of mRNA Expression of NRF2, cGAS, STING, and NF-κB in Each Group. The primer sequences used in this study are listed in Table 1.

#### 2.2.7. Western Blot (WB) Detection of NRF2, cGAS, STING, and NF-κB Protein Expression in Each Group

Total protein was extracted from cells of each group using RIPA lysis buffer containing PMSF. Protein concentration was determined using a BCA protein assay kit. Equal amounts of protein (50 μg) from each group were separated by sodium dodecyl sulfate–polyacrylamide gel electrophoresis (SDS-PAGE) under the following conditions: 200 V for 40 min. After electrophoresis, proteins were transferred onto a nitrocellulose (NC) membrane via the wet transfer method at a constant current of 300 mA for 40 min. The membrane was then blocked with 5% skim milk solution on a shaker at room temperature for 2 h. Following blocking, the membrane was incubated with the following primary antibodies diluted as specified: GAPDH (1:5000), NRF2 (1:1000), cGAS (1:2000), STING (1:20,000), and NF-κB (1:5000). After washing with TBST buffer, the membrane was incubated with species-corresponding HRP-conjugated secondary antibodies: Goat Anti-Rat IgG Antibody (1:15,000) and Goat Anti-Rabbit IgG Antibody (1:13,000) at room temperature for 1–2 h. Following TBST washes, the membrane was reacted with a hypersensitive ECL chemiluminescent substrate. Protein bands were visualized and imaged using a gel imaging analysis system. Relative band intensities of the target proteins were analyzed using ImageJsoftware (Version 1.8.0, National Institutes of Health, Bethesda, MD, USA).

#### 2.2.8. Immunofluorescence Localization of NRF2, cGAS, STING, and NF-κB Protein Expression

Cells were seeded in 12-well plates at a density of 1 × 10^6^ cells per well and cultured in a 37 °C, 5% CO_2_ incubator for 24 h. Different concentrations of drugs were then added, and incubation continued for another 24 h. The cell slides were washed three times with PBS for 3–5 min each time. The cells were fixed with 400 μL of pre-cooled 4% paraformaldehyde at room temperature for 15–30 min, followed by three PBS washes (3–5 min each). The slides were then permeabilized with 400 μL of 0.2% Triton X-100 (prepared in PBS) at 37 °C for 5–20 min, and again washed three times with PBS (3–5 min each). Residual PBS was removed with absorbent paper, and 400 μL of 5% goat serum was applied to the slides for blocking at 37 °C for 30 min.

After removing the blocking solution with absorbent paper, 200 μL of primary antibody was added to each slide, and the slides were placed in a humidified chamber for overnight incubation at 4 °C. Primary antibodies were diluted as follows: NRF2 (1:100), cGAS (1:200), STING (1:100), and NF-κB (1:100). The slides were then washed three times with PBS for 3–5 min each. After removing excess liquid with absorbent paper, 40 μL of diluted fluorescent secondary antibody was applied, and the slides were incubated in a humidified chamber at room temperature for 1 h. The secondary antibody was then recovered, and the slides were washed three times with PBS (3–5 min each). Residual liquid was removed with absorbent paper, and a drop of anti-fade mounting medium containing DAPI was placed on a glass slide. The cell slides were carefully removed using forceps and a needle and inverted onto the mounting medium. The edges were sealed with nail polish.

In this section, where applicable, authors are required to disclose details of how generative artificial intelligence (GenAI) has been used in this paper (e.g., to generate text, data, or graphics, or to assist in study design, data collection, analysis, or interpretation). The use of GenAI for superficial text editing (e.g., grammar, spelling, punctuation, and formatting) does not need to be declared.

## 3. Results

### 3.1. Effect of Curcumin (CUR) on TNF-Induced Proliferation of MH7A Cells

**Figure 1:**
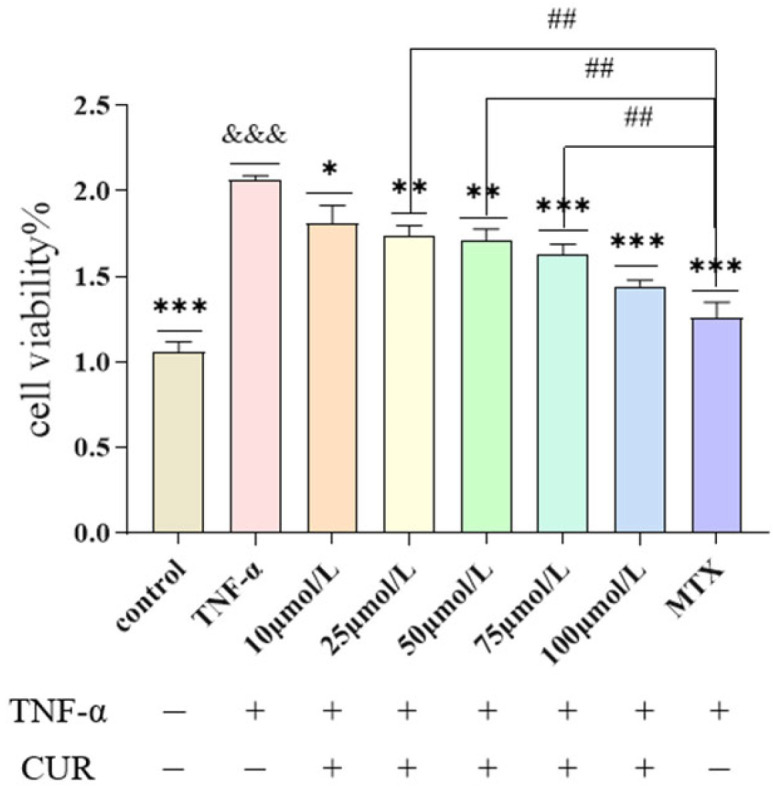
Effect of Curcumin on TNF-Induced MH7A Cell Proliferation. The figure shows the effect of curcumin on TNF-induced MH7A cell viability as detected by the CCK-8 assay. Compared to the control group, &&& *p* < 0.001, Compared with the TNF group, *** *p* < 0.001, ** *p* < 0.01, * *p* < 0.05; compared with the MTX group, ## *p* < 0.01.

Compared with the Control group, cell viability in the TNF (20 ng/mL) group was significantly increased after 24 h of intervention (*p* < 0.001). Compared with the TNF group, cell viability in the MTX group was markedly decreased (*p* < 0.001). After 24 h of treatment with different concentrations of curcumin, cell viability in each curcumin-treated group showed a dose-dependent decreasing trend (*p* < 0.05). Furthermore, compared with the MTX (positive drug control) group, cell viability in the CUR groups at concentrations of 25, 50, and 75 μmol/L was significantly higher (*p* < 0. 05). The experimental results are shown in Figure 1.

### 3.2. Effect of Curcumin (CUR) on TNF-Induced Migration of MH7A Cells

**Figure 2:**
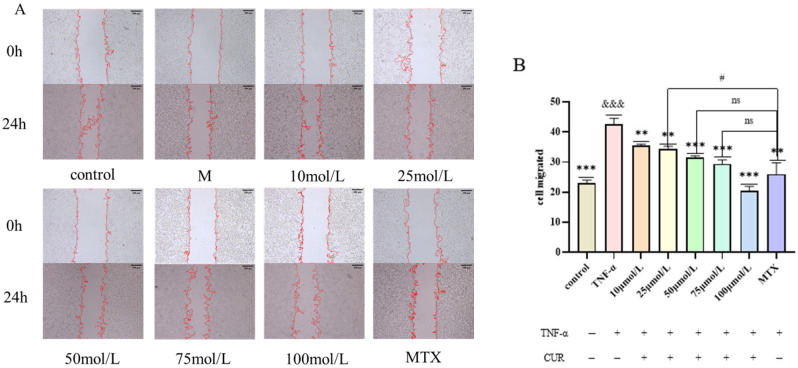
Effect of Curcumin on TNF -Induced Migration of MH7A Cells. (**A**) shows the migration status of cells in each group after 24 h, and (**B**) shows the migration rate of each group. Compared to the control group, &&& *p* < 0.001, compared with the TNF group, *** *p* < 0.001, ** *p* < 0.01; compared with the MTX group, # *p* < 0.05. ns = not significant.

Compared with the Control group, the cell migration rate in the TNF (20 ng/mL) group was significantly increased after 24 h of intervention (*p* < 0.001). Compared with the TNF group, the migration rate in the MTX group was markedly decreased (*p* < 0.01). After 24 h of treatment with different concentrations of curcumin, the cell migration rate in each CUR-treated group showed a dose−dependent and highly significant decreasing trend (*p* < 0.001). In comparison with the MTX (positive drug) group, the migration rate in the 25 μmol/L CUR group was significantly higher (*p* < 0.05), while no significant difference was observed in the migration rates of the 50 μmol/L and 75 μmol/L CUR groups. The experimental results are shown in Figure 2.

### 3.3. Effect of Curcumin (CUR) on TNF-Induced Invasion of MH7A Cells

**Figure 3:**
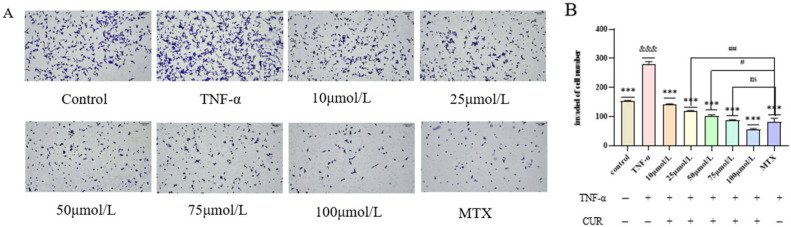
Effect of Curcumin on TNF-Induced MH7A Cell Invasion. (**A**) shows the invasion images of cells in each group, and (**B**) shows the number of invasive cells in each group. Compared to the control group, &&& *p* < 0.001, compared with the TNF group, *** *p* < 0.001; compared with the MTX group, ## *p* < 0.01, # *p* < 0.05. ns = not significant.

Compared with the Control group, the number of invasive cells in the TNF (20 ng/mL) group was significantly increased after 24 h of intervention (*p* < 0.001). Compared with the TNF group, the number of invasive cells in the MTX group was markedly reduced (*p* < 0.001). After treatment with different concentrations of CUR for 24 h, the number of invasive cells in each CUR-treated group showed a highly significant dose-dependent decrease (*p* < 0.001). Compared with the MTX (positive drug) group, the number of invasive cells was significantly higher in the 25 μmol/L CUR group (*p* < 0.01) and in the 50 μmol/L CUR group (*p* < 0.05), while no significant difference was observed in the 75 μmol/L CUR group. The experimental results are shown in Figure 3.

### 3.4. Effect of Curcumin (CUR) on TNF-Induced Apoptosis in MH7A Cells

**Figure 4:**
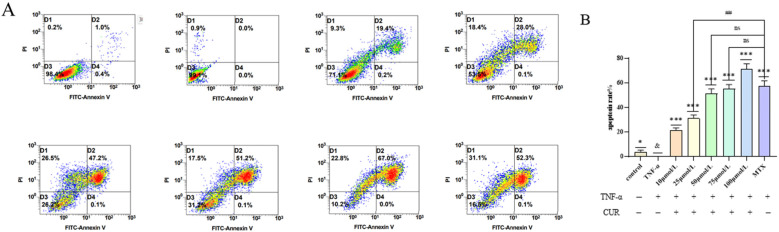
Effect of Curcumin on TNF-Induced Apoptosis in MH7A Cells. (**A**) shows the flow cytometry results of Annexin V/PI double-staining for each group, and (**B**) shows the apoptosis rate of each group. Compared to the control group, & *p* < 0.05, compared with the TNF group: *** *p* < 0.001, * *p* < 0.05; compared with the MTX group: ## *p* < 0.01, ns = not significant. Different colors represent cell density: red indicates the highest cell density, green is intermediate, and blue represents the lowest cell density.

Compared with the Control group, the apoptosis rate in the TNF (20 ng/mL) group was significantly decreased after 24 h of intervention. Compared with the TNF group, the apoptosis rate in the MTX group was markedly increased to 52.4% (*p* < 0.01). After 24 h of treatment with different concentrations of CUR, the apoptosis rate showed an increasing trend with higher drug concentrations, reaching a statistically significant difference at 25 μmol/L (*p* < 0.01). In comparison with the MTX (positive drug) group, no significant difference in apoptosis rate was observed in the 50 μmol/L and 75 μmol/L CUR groups. The experimental results are shown in Figure 4.

### 3.5. mRNA Expression Levels of NRF2, cGAS, STING, and NF-κB in Each Group

**Figure 5:**
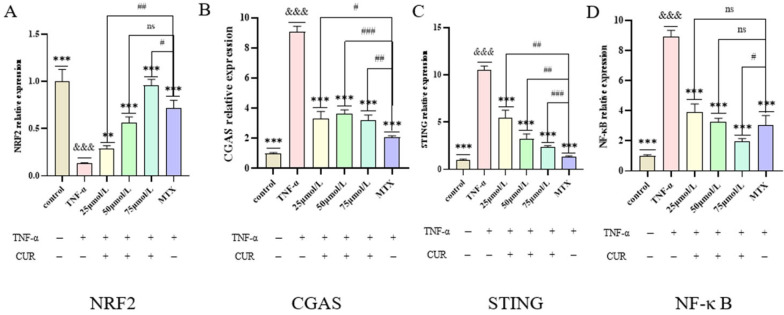
mRNA Expression Levels of NRF2, cGAS, STING, and NF-κB in Each Group. (**A**–**D**) represent the mRNA expression levels of NRF2, cGAS, STING, and NF-κB in each group, respectively. Compared to the control group, &&& *p* < 0.001, compared with the TNF group: *** *p* < 0.001, ** *p* < 0.01; compared with the MTX group: ### *p* < 0.001, ## *p* < 0.01, # *p* < 0.05, ns = not significant.

The qRT-PCR results showed that, compared with the Control group, the expression of NRF2 mRNA in the TNF-induced group was significantly decreased (*p* < 0.001), while the mRNA levels of cGAS, STING, and NF-κB were significantly increased (*p* < 0.001). Compared with the TNF-induced group, both the MTX group and the CUR groups exhibited significantly increased NRF2 mRNA expression (*p* < 0.01) and significantly decreased mRNA levels of cGAS, STING, and NF-κB. The experimental results are shown in Figure 5.

### 3.6. Protein Expression Levels of NRF2, cGAS, STING, and NF-κB in Each Group

**Figure 6:**
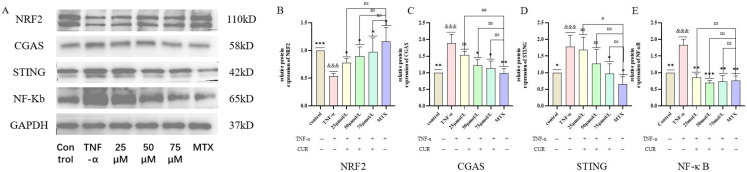
Protein Expression Results of NRF2, cGAS, STING, and NF-κB in Each Group. Note: (**A**) shows the Western Blot images of NRF2, cGAS, STING, and NF-κB in each group; (**B**–**E**) represent the protein expression results of NRF2, cGAS, STING, and NF-κB, respectively, in each group. Compared to the control group, &&& *p* < 0.001, compared with the TNF group: *** *p* < 0.001, ** *p* < 0.01, * *p* < 0.05; compared with the MTX group: ## *p* < 0.01, # *p* < 0.05, ns = not significant.

The protein expression levels of NRF2, cGAS, STING, and NF-κB in each group showed that, compared with the Control group, the expression of NRF2 protein in the TNF-induced group was significantly decreased (*p* < 0.001), while the protein expression levels of cGAS, STING, and NF-κB were significantly increased (*p* < 0.001). Compared with the TNF-induced group, both the MTX group and the CUR groups exhibited significantly increased NRF2 protein expression (*p* < 0.01 or *p* < 0.001) and significantly decreased protein expression levels of cGAS, STING, and NF-κB. The experimental results are shown in Figure 6.

### 3.7. Immunofluorescence Localization of NRF2, cGAS, STING, and NF-κB Protein Expression

**Figure 7:**
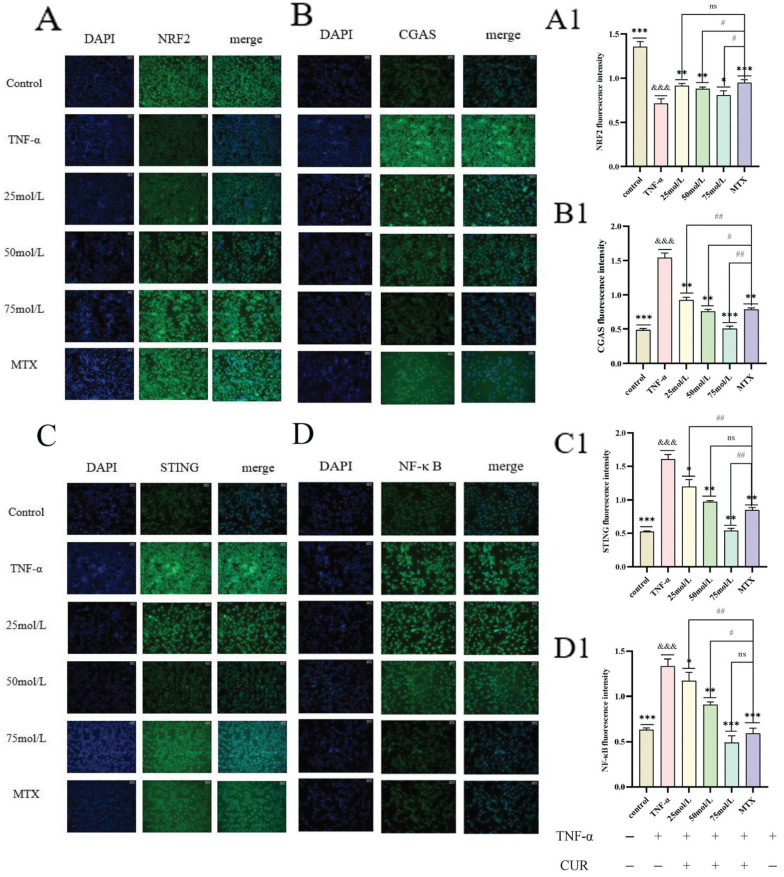
Localization of NRF2, cGAS, STING, and NF-κB Proteins in Each Group. (**A**–**D**) show immunofluores-cence images of NRF2, cGAS, STING, and NF-κB, respectively, in each group. (**A1**–**D1**) represent the quantitative fluorescence intensity of NRF2, cGAS, STING, and NF-κB, respectively, in each group. Compared to the control group, &&& *p* < 0.001 compared with the TNF group: *** *p* < 0.001, ** *p* < 0.01, * *p* < 0.05; compared with the MTX group: ## *p* < 0.01, # *p* < 0.05, ns = not significant.

The immunofluorescence expression levels of NRF2, cGAS, STING, and NF-κB in each group showed that, compared with the Control group, the fluorescence intensity of NRF2 in the TNF-induced group was significantly decreased (*p* < 0.001), while the fluorescence intensities of cGAS, STING, and NF-κB were significantly increased (*p* < 0.001). Compared with the TNF-induced group, both the MTX group and the CUR groups exhibited significantly increased NRF2 fluorescence intensity (*p* < 0.01 or *p* < 0.001) and significantly decreased fluorescence intensities of cGAS, STING, and NF-κB. The experimental results are shown in Figure 7.

## 4. Discussion

Rheumatoid arthritis (RA) is an autoimmune disease characterized by chronic inflammation of the synovial membrane and abnormal proliferation of fibroblasts. During the pathological progression of RA, the abnormal activation, invasion, and apoptosis-resistant “tumor-like” phenotype of synovial fibroblasts (FLS) are the core drivers of progressive joint destruction [18]. Current RA treatment still faces challenges in achieving both effective inflammation control and targeted reversal of the destructive phenotype of synovial fibroblasts (FLS). Therefore, exploring natural product-derived candidate molecules capable of multi-target intervention in RA pathological processes with improved safety profiles holds significant value. Curcumin, a natural polyphenol with a long history of medicinal use, has been widely recognized for its anti-inflammatory and antioxidant activities. However, its precise molecular network in RA, particularly regarding the malignant phenotype of FLS, still requires in-depth elucidation.

At the cellular level, this study confirmed that curcumin can effectively reverse the activation state of human RA synovial fibroblasts (MH7A) induced by tumor necrosis factor-α (TNF) [19,20]. The results showed that with increasing concentrations, curcumin significantly reduced the migration and invasion ability of MH7A cells and markedly increased the apoptosis rate, indicating that curcumin inhibits the migration and invasion of MH7A cells and promotes their apoptosis in a concentration-dependent manner. This is consistent with findings from Kloesch B et al. [21], further supporting the view that curcumin can restrain the destructive behavior of RA-FLS and reverse their apoptotic deficiency. Notably, the inhibitory effect of curcumin on cell proliferation was weaker than that of MTX within a certain concentration range, suggesting that its mode of action may not involve strong cytotoxicity but rather multifaceted modulation of cellular homeostasis.

This study investigated the molecular mechanism by which curcumin (CUR) intervenes in rheumatoid arthritis (RA) through regulating the NRF2-cGAS-STING-NF-κB cascade pathway. In the pathological process of RA, DNA damage induced by oxidative stress and mitochondrial dysfunction mediated by TNF can both lead to the abnormal accumulation of cytoplasmic double-stranded DNA (mtDNA). This nucleic acid molecule is recognized by the key DNA sensor cGAS, which in turn catalyzes the generation of the second messenger cGAMP and activates the downstream STING signaling axis. Clinical evidence indicates that STING expression is significantly upregulated in the joint tissues of RA patients, while the activity of the antioxidant transcription factor NRF2 is inhibited; the degree of this imbalance is closely related to disease activity and joint destruction. From a functional regulatory perspective, NRF2 exerts anti-inflammatory effects by inhibiting the activation of NF-κB and the release of downstream pro-inflammatory factors (such as IL-6, TNF-α). Meanwhile, although STING signaling primarily promotes inflammation, it can also play a negative immunoregulatory role at specific stages by modulating B cell function. Notably, both the NRF2 and STING pathways ultimately converge on the inhibition of NF-κB activity, which provides a reasonable explanation for the pathway’s effects on inhibiting RA synovial cell proliferation and promoting its apoptosis.

The core finding of this study lies in linking the protective effect of curcumin on RA-FLS to the regulation of the emerging inflammatory-immune axis “NRF2-cGAS-STING-NF-κB”. In the pathological microenvironment of RA, sustained oxidative stress and inflammation can lead to abnormal intracellular accumulation of double-stranded DNA (dsDNA), including mitochondrial DNA (mtDNA) [13,22,23]. Experimental results showed that after TNF induction, both the mRNA and protein expression levels of NRF2 in MH7A cells were significantly decreased, confirming that TNF stimulation markedly suppresses NRF2 expression while activating the cGAS-STING-NF-κB pathway [24,25]. Notably, the study by Maria [26] has clearly demonstrated that curcumin activates the endogenous antioxidant defense system via the Nrf2 pathway, markedly elevates the synthesis of antioxidant substances such as glutathione, and efficiently scavenges intracellular reactive oxygen species (ROS). This effect precisely provides a key molecular target for curcumin to block the ROS-mediated activation of the cGAS-STING-NFκB pathway in rheumatoid arthritis (RA). In contrast, curcumin treatment exhibited an opposite regulatory pattern: it significantly upregulated the expression of NRF2, a master regulator of cellular defense, and simultaneously inhibited the activation of its downstream factors cGAS, STING, and NF-κB. This was reflected by a significant increase in NRF2 mRNA and protein levels and a significant decrease in cGAS, STING, and NF-κB mRNA and protein levels in curcumin-treated groups. These results strongly suggest that curcumin may enhance cellular antioxidant capacity by activating NRF2, thereby reducing oxidative stress-induced DNA damage and subsequent dsDNA leakage [27]. Concurrently, NRF2 activation may directly or indirectly interfere with cGAS recognition of dsDNA or STING signal transduction [14,28], ultimately blocking the activation of downstream NF-κB and type I interferon pathways. As a central transcription factor in inflammatory responses, the inhibition of NF-κB activity directly explains the observed anti-inflammatory, anti-proliferative, and pro-apoptotic effects of curcumin [29,30,31]. Therefore, the mechanism of curcumin can be summarized as follows: by empowering NRF2, an endogenous defense system, it mitigates cellular stress at the source, thereby suppressing excessive innate immune responses triggered by aberrant DNA sensing (cGAS-STING) and the classical inflammatory pathway (NF-κB), Genetic predisposition affecting specific immune cells can exert a causal effect on the risk of developing rheumatoid arthritis (RA) [5,32]. This provides further evidence for the critical role of the immune system in the pathogenesis of the disease, forming a multi-layered synergistic inhibitory network.

Comparison with the clinically commonly used drug methotrexate (MTX) revealed that, although curcumin demonstrated effects comparable to MTX in inducing apoptosis and inhibiting invasion at higher concentrations, its efficacy in suppressing proliferation and migration was lower than that of MTX within certain concentration ranges [2,25]. This precisely highlights the characteristic of curcumin as a multi-target natural compound: rather than acting through a single, potent cytotoxic mechanism, it exerts a mild and synergistic modulation of the continuous signaling network “NRF2-cGAS-STING-NF-κB” to reshape cellular homeostasis and systemically reverse the pathogenic state of FLS. This mode of action may imply a better safety profile and provides a theoretical basis for combination therapy with existing drugs to reduce respective doses and minimize side effects.

Although natural curcumin used in the present study can effectively regulate the Nrf2-cGAS-STING-NF-κB pathway, its therapeutic efficacy in vivo is still limited by low oral bioavailability, leaving room for further improvement. At the mechanistic level, gene editing techniques can be employed to verify the causal relationship of the pathway: the CRISPR/Cas9 system can be utilized to knockdown or overexpress Nrf2 in MH7A cells, so as to detect whether the regulatory effect of curcumin on the cGAS-STING-NF-κB pathway is abrogated after Nrf2 knockdown and whether the inhibitory effect of curcumin is potentiated following Nrf2 overexpression, thus directly validating the upstream core regulatory role of Nrf2 in this pathway. Meanwhile, cGAS and STING can be knocked down to examine whether the activation of Nrf2 by curcumin is affected, so as to clarify the upstream and downstream regulatory relationships within the pathway. At the translational application level, based on the findings reported in Maria [26,32], subsequent studies can be designed to develop curcumin derivatives or nanodelivery systems to enhance the targeted activation efficiency of the Nrf2 pathway in RA synovial tissue, further strengthen the inhibitory effect on the cGAS-STING-NF-κB pathway, and provide optimized strategies for the clinical translation of curcumin.

Several limitations of this study should be noted. First, all conclusions of this study are based on in vitro cell experiments, and no cell co-culture model was established, with only the responses of fibroblast-like synoviocytes investigated in isolation. Although the MH7A cell line is a classic model for studying rheumatoid arthritis fibroblast-like synoviocytes (RA-FLS), it cannot fully replicate the complex crosstalk between fibroblast-like synoviocytes and immune-related cells such as macrophages, T cells and chondrocytes in the in vivo joint microenvironment, nor can it recapitulate the in vivo pharmacokinetic characteristics and the actual joint microenvironment status. Therefore, it is difficult to reflect the anti-inflammatory effects and pathway regulatory features of curcumin in the complex immune microenvironment.

In summary, this study demonstrates that curcumin effectively inhibits inflammatory activation, migration, and invasion while promoting apoptosis in RA synovial fibroblasts by activating NRF2 and suppressing the downstream cGAS-STING-NF-κB signaling cascade. This finding not only provides a novel molecular-biological explanation for the traditional therapeutic effects of curcumin in RA, linking three key pathological aspects—“antioxidant defense (NRF2),” “innate immune sensing (cGAS-STING),” and “classical inflammation (NF-κB)”—into an integrated framework, but also lays an important experimental foundation for developing novel RA treatment strategies targeting this pathway, which may offer the advantages of multi-target modulation and a favorable safety profile. Future studies should validate this pathway in animal models (e.g., CIA mice) and employ gene-editing techniques to clarify its causal relationships, thereby advancing the translation of curcumin from a natural product to a mechanism-based potential anti-rheumatic drug.

## Figures and Tables

**Table 1:** Primer sequence information.

Gene	Genbank	Sequence (5′→3′)	MW/bp
*NRF2*	NM_001145412.3	F: TTCTCCCAATTCAGCCAGCC	145
*cGAS*	NM_001410911.1	F: GAAGAAACATGGCGGCTATC R: GAGGGTTCTGGGTACATACG	87
*STING*	NM_001301738.2	F: CAGGCACTGAACATCCTCCT R: ATATACAGCCGCTGGCTCAC	146
*NF-Κb*	NM_001382627.1	F: GGAGACGTGAAGATGCTGCT R: GGGTGTGTTCCATCGTAGG	238
*GAPDH*	NM_017008.4	F: ATGACTCTACCCACGGCAAG R: TGGGTTTCCCGTTGATGACC	75

## Data Availability

Data are available from the corresponding author upon request.

## References

[1] Serhal L., Lwin M.N., Holroyd C., Edwards C.J. (2020). Rheumatoid arthritis in the elderly: Characteristics and treatment considerations. Autoimmun. Rev..

[2] Zhao Z., Hua Z., Luo X., Li Y., Yu L., Li M., Lu C., Zhao T., Liu Y. (2022). Application and pharmacological mechanism of methotrexate in rheumatoid arthritis. Biomed. Pharmacother..

[3] Brown P., Pratt A.G., Hyrich K.L. (2024). Therapeutic advances in rheumatoid arthritis. BMJ.

[4] Grötsch B., Bozec A., Schett G. (2025). Models of Rheumatoid Arthritis. Methods Mol. Biol..

[5] El Shebiny E., Shoeib S., Moeen S.M., Badr E., Elshabacy F., Meiz E., Zahran E. (2024). Serum Hepcidin: An Atherosclerotic Biomarker in Rheumatoid Arthritis Patients: A multicenter Case-Control Study. Eurasian J. Med. Oncol..

[6] Wang Y., Chen S., Du K., Liang C., Wang S., Owusu Boadi E., Li J., Pang X., He J., Chang Y.X. (2021). Traditional herbal medicine: Therapeutic potential in rheumatoid arthritis. J. Ethnopharmacol..

[7] Li N., Li X., Deng L., Yang H., Gong Z., Wang Q., Pan D., Zeng S., Chen J. (2023). 6-Shogaol inhibits the proliferation, apoptosis, and migration of rheumatoid arthritis fibroblast-like synoviocytes via the PI3K/AKT/NF-κB pathway. Phytomedicine.

[8] Zhou S., Huang G. (2022). Some important inhibitors and mechanisms of rheumatoid arthritis. Chem. Biol. Drug Des..

[9] Kou H., Huang L., Jin M., He Q., Zhang R., Ma J. (2023). Effect of curcumin on rheumatoid arthritis: A systematic review and meta-analysis. Front. Immunol..

[10] Pourhabibi-Zarandi F., Shojaei-Zarghani S., Rafraf M. (2021). Curcumin and rheumatoid arthritis: A systematic review of literature. Int. J. Clin. Pract..

[11] Deng T., Xu J., Wang Q., Wang X., Jiao Y., Cao X., Geng Q., Zhang M., Zhao L., Xiao C. (2024). Immunomodulatory effects of curcumin on macrophage polarization in rheumatoid arthritis. Front. Pharmacol..

[12] Liu C., Rokavec M., Huang Z., Hermeking H. (2023). Curcumin activates a ROS/KEAP1/NRF2/miR-34a/b/c cascade to suppress colorectal cancer metastasis. Cell Death Differ..

[13] Sun L., Niu Y., Liao B., Liu L., Peng Y., Li K., Chen X., Chen Q., Bai D. (2025). CUR-PDT induces ferroptosis of RA-FLS via the NRF2/xCT/GPX4 pathway to inhibit proliferation in rheumatoid arthritis. Inflamm. Res..

[14] Xu Y., Wang L., Liao H., Li X., Zhang Y., Chen X., Xu B., Liu Y., Tu W., Liu Y. (2024). Loss of NRF2 aggravates ionizing radiation-induced intestinal injury by activating the cGAS/STING pathway via Pirin. Cancer Lett..

[15] Samson N., Ablasser A. (2022). The cGAS-STING pathway and cancer. Nat. Cancer.

[16] Zeng L., Yang T., Yang K., Yu G., Li J., Xiang W., Chen H. (2022). Curcumin and curcuma longa extract in the treatment of 10 types of autoimmune diseases: A systematic review and meta-analysis of 31 randomized controlled trials. Front. Immunol..

[17] Han Y., Liu C., Chen S., Sun H., Jia Z., Shi J., Wang L., Du K., Chang Y. (2025). Columbianadin ameliorates rheumatoid arthritis by attenuating synoviocyte hyperplasia through targeted vimentin to inhibit the VAV2/Rac-1 signaling pathway. J. Adv. Res..

[18] Yang X., Zhao L., Pang Y. (2024). cGAS-STING pathway in pathogenesis and treatment of osteoarthritis and rheumatoid arthritis. Front. Immunol..

[19] Bugatti S., De Stefano L., Gandolfo S., Ciccia F., Montecucco C. (2023). Autoantibody-negative rheumatoid arthritis: Still a challenge for the rheumatologist. Lancet Rheumatol..

[20] Lin L., Huang Z., Li W., Liu X., Li X., Gao S., Chen J., Yang C., Min X., Yang H. (2024). Mid1 promotes synovitis in rheumatoid arthritis via ubiquitin-dependent post-translational modification. Pharmacol. Res..

[21] Kloesch B., Becker T., Dietersdorfer E., Kiener H., Steiner G. (2013). Anti-inflammatory and apoptotic effects of the polyphenol curcumin on human fibroblast-like synoviocytes. Int. Immunopharmacol..

[22] Cuadrado A., Manda G., Hassan A., Alcaraz M.J., Barbas C., Daiber A., Ghezzi P., León R., López M.G., Oliva B. (2018). Transcription Factor NRF2 as a Therapeutic Target for Chronic Diseases: A Systems Medicine Approach. Pharmacol. Rev..

[23] Wruck C.J., Fragoulis A., Gurzynski A., Brandenburg L.O., Kan Y.W., Chan K., Hassenpflug J., Freitag-Wolf S., Varoga D., Lippross S. (2011). Role of oxidative stress in rheumatoid arthritis: Insights from the NRF2-knockout mice. Ann. Rheum. Dis..

[24] Wang Y., Li Y., Jiang J., Hong Y., Gao S., Hua C. (2025). Ferritinophagy in inflammatory and autoimmune diseases: Mechanistic insights and therapeutic potentials. Autoimmun. Rev..

[25] Decout A., Katz J.D., Venkatraman S., Ablasser A. (2021). The cGAS-STING pathway as a therapeutic target in inflammatory diseases. Nat. Rev. Immunol..

[26] Martu M.-A., Maftei G.-A., Luchian I., Stefanescu O.M., Scutariu M.M., Solomon S.M. (2021). The Effect of Acknowledged and Novel Anti-Rheumatic Therapies on Periodontal Tissues—A Narrative Review. Pharmaceuticals.

[27] Wang W., Zhou H., Liu L. (2018). Side effects of methotrexate therapy for rheumatoid arthritis: A systematic review. Eur. J. Med. Chem..

[28] Yuan Q., Tang B., Zhang C. (2022). Signaling pathways of chronic kidney diseases, implications for therapeutics. Signal Transduct. Target. Ther..

[29] Blaj L.A., Cucu A.I., Tamba B.I., Turliuc M.D. (2023). The Role of the NF-kB Pathway in Intracranial Aneurysms. Brain Sci..

[30] de Vries C., Huang W., Sharma R.K., Wangriatisak K., Turcinov S., Cîrciumaru A., Rönnblom L., Grönwall C., Hensvold A., Lundberg K. (2025). Rheumatoid Arthritis Related B-Cell Changes Are Found Already in the Risk-RA Phase. Eur. J. Immunol..

[31] He X., Wang C., Zhang R., Wang Y., Zhang Y., Yang T., Zhang J., Rao S., Tang H., Peng X. (2026). Parthenolide ameliorates inflammation in sepsis via covalently targeting Trim33 and inhibiting NF-κB pathway. Phytomedicine.

[32] Peng Z., Huang W., Tang M., Chen B., Yang R., Liu Q., Liu C., Long P. (2024). Investigating the shared genetic architecture between hypothyroidism and rheumatoid arthritis. Front. Immunol..

